# A systems biology approach to cGMP suggests a prominent role for sGC in stroke: Validation by mechanism-based activation of apo-sGC in non-steal dosing coveys neuroprotection and increased survival

**DOI:** 10.1186/2050-6511-16-S1-A39

**Published:** 2015-09-02

**Authors:** Ana I Casas, Friederike Langhauser, Vu Thao-Vi Dao, Emre Guney, Pamela WM Kleikers, Manuela García López, Jörg Menche, Albert-László Barabási, Christoph Kleinschnitz, Harald HHW Schmidt

**Affiliations:** 1 Department of Pharmacology, CARIM, and Maastricht Institute for Advanced Studies, Maastricht University, Maastricht, the Netherlands; 2 Departamento de Farmacologia, Facultad de Medicina, Universidad Autónoma de Madrid, Madrid, Spain; 3 Department of Neurology, University Hospital Würzburg, Würzburg, Germany; 4 Center for Complex Network Research (CCNR) and Department of Physics, Northeastern University, Boston, MA 02115, USA

## 

Based on non-hypothesis-driven approaches genetic evidence suggests that diseases are interrelated differently to our current organ-based ontology. In fact, common effector mechanisms, when affected or triggered seem to produce pathophenotypes in diverse organs or co-morbidities. This will lead eventually to a revised disease nomenclature and opens up entirely new approaches for diagnosis and treatment. In this context, we noted that a common cardiovascular target, the cGMP-forming Fe(II) haem protein, soluble guanylate cyclase (sGC), appears to be situated in a common mechanism network that is prominently relevant to stroke. Ischemic stroke is the second leading cause of death worldwide and the leading cause of disability. Despite this high medical need only a single drug is available but due to its limited time window and risk of bleeding 85% of all patients are excluded from treatment. As a possible add-on, vasoactive drugs however typically dilate normal blood vessels and cause a paradoxical “steal phenomenon” by both shunting blood to healthy vascular beds and a systemic blood pressure drop. Upon middle cerebral artery occlusion sGC protein and nitric oxide-stimulated activity in the ischemic hemisphere were dramatically down-regulated leading to a high proportion of oxidized and/or haem-free apo-sGC activity. Pharmacological targeting of apo-sGC in vitro under oxygen and glucose deprivation conveyed strong neuroprotection via ERK/CREB signalling. In vivo, post-stroke apo-sGC activation by two distinct members of this compound class augmented cerebral blood-flow whilst leaving systemic blood pressure unaffected, reduced infarct size and increased survival. Different apo-sGC activators are in advanced stages of clinical development for different cardiovascular indications. Systems biology and network medicine and our preliminary target validation suggest that they should be urgently tested for repurposing as first-in-class, mechanism-based neuroprotective drugs in stroke.

